# Right- and left-sided colorectal cancers respond differently to cetuximab

**DOI:** 10.1186/s40880-015-0022-x

**Published:** 2015-06-10

**Authors:** Feng Wang, Long Bai, Tian-Shu Liu, Yi-Yi Yu, Ming-Ming He, Kai-Yan Liu, Hui-Yan Luo, Dong-Sheng Zhang, Yin Jin, Feng-Hua Wang, Zhi-Qiang Wang, De-Shen Wang, Miao-Zhen Qiu, Chao Ren, Yu-Hong Li, Rui-Hua Xu

**Affiliations:** Department of Medical Oncology, Sun Yat-sen University Cancer Center: State Key Laboratory of Oncology in South China; Collaborative Innovation Center for Cancer Medicine, 651 Dongfeng Road East, Guangzhou, Guangdong 510060 P. R. China; Department of Medical Oncology, Zhongshan Hospital, Fudan University, Shanghai, 200032 P. R. China

**Keywords:** Colorectal neoplasms, Cetuximab, Left-sided, Right-sided, Chemotherapy

## Abstract

**Introduction:**

Right-sided colon cancer (RSCC) and left-sided colorectal cancer (LSCRC) differ with respect to their biology and genomic patterns. This study aimed to examine whether the primary tumor location is associated with the response to cetuximab in patients with metastatic colorectal cancer (mCRC).

**Methods:**

Patients with mCRC treated with cetuximab and standard chemotherapy as first- or second-line treatments were compared with randomly chosen patients who were treated with chemotherapy alone between 2005 and 2013. The main outcome measures were the overall response rate (ORR), progression-free survival (PFS), and overall survival (OS). The differences in the outcome were analyzed by using the chi-squared test, Student’s *t* test, and Kaplan-Meier method.

**Results:**

The treatment results of 206 patients with mCRC treated with cetuximab and standard chemotherapy as first- or second-line treatments were compared with those of 210 patients who were treated with chemotherapy alone. As a first-line treatment, cetuximab with chemotherapy was associated with a significantly higher ORR (49.4 % vs. 28.6 %, *P* = 0.005) as well as longer PFS (9.1 vs. 6.2 months, *P* = 0.002) and OS (28.9 vs. 20.1 months, *P* = 0.036) than chemotherapy alone in patients with LSCRC. However, cetuximab neither improved the ORR (36.4 % vs. 26.2 %, *P* = 0.349) nor prolonged PFS (5.6 vs. 5.7 months, *P* = 0.904) or OS (25.1 vs. 19.8 months, *P* = 0.553) in patients with RSCC. As a second-line treatment, cetuximab exhibited a tendency to improve the ORR (23.5 % vs. 10.2 %, *P* = 0.087) and prolong PFS (4.9 vs. 3.5 months, *P* = 0.064), and it significantly prolonged OS (17.1 vs. 12.4 months, *P* = 0.047) compared with chemotherapy alone in the patients with LSCRC. In contrast, as a second-line treatment, cetuximab neither improved the ORR (7.1 % vs. 11.4 %, *P* = 0.698) nor prolonged PFS (3.3 vs. 4.2 months, *P* = 0.761) or OS (13.4 vs. 13.0 months, *P* = 0.652) in patients with RSCC.

**Conclusions:**

The addition of cetuximab to chemotherapy in both first- and second-line treatments of mCRC may only benefit patients with primary LSCRC.

## Background

Colorectal carcinomas (CRCs) that occur proximal (right) or distal (left) to the splenic flexure exhibit differences in their embryologic development, blood supply, macroscopic pathology, and clinicopathologic parameters [1, 2]. The right colon arises from the embryonic midgut and is perfused by the superior mesenteric artery, whereas the left colon originates from the hindgut and is served by the inferior mesenteric artery [2]. Right-sided colon cancers (RSCCs) are typically bulky, exophytic, polypoid lesions that project into the lumen and cause significant anemia, whereas left-sided colorectal cancers (LSCRCs) are typically infiltrating, constricting lesions that encircle the lumen, often leading to obstruction [1]. Poorly differentiated adenocarcinoma or signet-ring cell carcinoma and mucinous adenocarcinoma are more frequently seen in the right colon than in the left colon [3]. RSCCs typically present at a more advanced stage, and the patients with RSCCs have a significantly worse survival than patients with LSCRCs [4–6].

Recent studies have revealed distinguishable genomic patterns between LSCRC and RSCC [7, 8]. LSCRCs exhibit higher p53 gene mutation and cyclooxygenase-2 expression rates with more common chromosomal instability [9–12]. RSCCs are generally diploid, and they exhibit higher rates of microsatellite instability (MSI) and higher expression of cytoplasmic c-erbB2 and epidermal growth factor receptors (EGFRs) [13]. Recent whole genome analysis has shown that RSCCs are more likely to be hypermethylated as well as to have elevated mutation rates compared with LSCRCs [7]. Some studies have reported significantly more activating mutations in codon 12/13 of Kirsten rat sarcoma viral oncogene homolog (*KRAS*) in RSCCs than in LSCRCs [14, 15]. However, other studies have reported that there is no substantial difference in the *KRAS* mutations between RSCCs and LSCRCs [8, 16]. LSCRCs exhibit a significant association between *KRAS* activation and distant organ metastasis, whereas RSCCs do not. Mutation of *KRAS* was found to be associated with a significantly poorer prognosis in patients with LSCRCs, but not in those with RSCCs [17].

Cetuximab is a chimeric IgG1 monoclonal antibody that binds to the extracellular domain of EGFR; also, it blocks ligand-induced receptor signaling and induces immune-mediated antitumor mechanisms, such as antibody-dependent cell-mediated cytotoxicity [18, 19]. Cetuximab is currently approved for the treatment of metastatic CRC with wild-type *KRAS* as a monotherapy or in combination with chemotherapy in first-, second-, or third-line settings [20]. Only patients with metastatic colorectal cancer (mCRC) expressing wild-type *KRAS* respond to cetuximab and show improvements in progression-free survival (PFS) and overall survival (OS) [21]. *KRAS* mutations and the B-Raf proto-oncogene, serine/threonine kinase (*BRAF*) V600E mutation, the key molecules of the epidermal growth factor (EGF) pathway, are predictors of resistance to cetuximab therapy [21–23].

It remains unknown whether the primary tumor location affects the response of mCRC patients to first- and second-line cetuximab treatments. This retrospective multicenter study aimed to investigate the association of the tumor location with tumor response to cetuximab and survival in mCRC patients.

## Patients and methods

### Patients and treatment

In this retrospective study, we assessed mCRC patients treated with cetuximab plus chemotherapy as a first- or second-line therapy at two national cancer centers (Sun Yat-sen University Cancer Center and Fudan University Cancer Center) between January 2006 and December 2013 (the cetuximab group). Similar numbers of patients with LSCRC and RSCC who received first- or second-line chemotherapy without cetuximab during the same period were included as the chemotherapy alone group by means of isometric mechanical random sampling as control groups. Enrolled patients have received mFOLFOX-6 (oxaliplatin 85 mg/m^2^ d1, 5-fluorouracil [5-FU] bolus 400 mg/ m^2^ d1, 5-FU 2,400 mg/m^2^ continuous infusion for 46 hours every 2 weeks), XELOX (oxaliplatin 130 mg/m^2^ d1, capecitabine 2,000 mg/m^2^ d1–14 every 3 weeks), or modified FOLFIRI (irinotecan 180 mg/m^2^ d1, 5-FU bolus 400 mg/m^2^ d1, 5-FU 2,400 mg/m^2^ continuous infusion for 46 hours every 2 weeks) and in combination with 5-FU–based regimens (cetuximab 400 mg/m^2^ taken at the first dose and followed by 500 mg/m^2^ every 2 weeks) or capecitabine-based regimens (capecitabine 750 mg/m^2^ every 3 weeks) in the cetuximab group. All patients had histologically proven mCRC and offered written informed consent for possible future data analysis before treatment. Colon cancers arising in or proximal to the splenic flexure were defined as RSCCs, and those arising distal to the splenic flexure were defined as LSCRCs. Clinicpathologic characteristics (patient age, sex, performance status, and pathologic subtype), curative-intent metastasis resection, treatment duration of chemotherapy, and cetuximab, backbone chemotherapy regimen, exposure to three active chemotherapy agents, exposure to bevacizumab therapy, disease-free survival after curative-intent primary tumor resection, and follow-up data were compared between patients with LSCRCs and those with RSCCs. The tumor response was evaluated by computerized tomodensitometry according to the Response Evaluation Criteria in Solid Tumors (RECIST) version 1.1. Objective response rate (ORR) was defined as percentage of patients showing complete response (CR) or partial response (PR) as best response according to RECIST.

### Statistical analysis

The patient characteristics and response rates were compared by using Student’s *t* test or the chi-square test. The PFS was calculated from the initiation of a first- or second-line treatment to the date of tumor progression, death from any cause, or the last follow-up. The OS was defined as the duration from the start of a first- or second-line therapy to death of any cause or the date of the last follow-up. All point data were censored. Both the PFS and OS were estimated by using the Kaplan-Meier method and compared by using the log-rank test. Statistical analysis was performed by using SPSS software 19.0 (SPSS Inc., Chicago, IL, USA). The level of significance was set at *P* = 0.05.

## Results

### Patient characteristics

A total of 206 mCRC patients who received combination treatment with cetuximab and chemotherapy as a first-line (110 patients) or second-line (96 patients) therapy were included in this study. In addition, 210 mCRC patients were chosen as controls from those who received first-line (117 patients) or second-line chemotherapy (93 patients) without receiving cetuximab during the same period. The clinicopathologic characteristics of these patients are shown in Tables 1 and 2. In patients who received first-line therapy, all characteristics except the backbone chemotherapy were well-balanced between the cetuximab group and the chemotherapy alone group (Table 1).

**Table 1:** Baseline clinicopathologic characteristics of patients with first-line treatment

Clinicopathologic characteristic		Cetuximab group					Chemotherapy alone group				*P* value ^d^
		All patients	RSCC	LSCRC	*P* value		All patients	RSCC	LSCRC	*P* value	
No. of patients		110	33	77	–		117	61	56	–	0.001*
Median age at diagnosis					0.420					0.502	0.613
≤65 years		90 (81.8)	29 (87.9)	62 (80.5)			93 (79.5)	50 (82.0)	43 (76.8)		
>65 years		20 (18.2)	4 (12.1)	15 (19.5)			24 (20.5)	11 (18.0)	13 (23.2)		
Sex					0.274					0.085	0.404
Male		75 (68.2)	20 (60.6)	55 (71.4)			73 (62.4)	43 (70.5)	30 (53.6)		
Female		35 (31.8)	13 (39.4)	22 (28.6)			44 (37.6)	18 (29.5)	26 (46.4)		
ECOG performance status					0.300					0.367	0.113
0		49 (44.5)	11 (33.3)	38 (49.4)			49 (41.9)	29 (47.5)	20 (35.7)		
1		58 (52.7)	21 (63.6)	37 (48.1)			57 (48.7)	26 (42.6)	31 (55.4)		
≥2		3 (2.7)	1 (3.0)	2 (2.6)			11 (9.4)	6 (9.8)	5 (8.9)		
Pathology					0.003*					0.116	0.608
Adenocarcinoma		101 (91.8)	26 (78.8)	75 (97.4)			110 (94.0)	55 (90.2)	55 (98.2)		
Mucinous & signet-ring cell		9 (8.2)	7 (21.2)	2 (2.6)			7 (6.0)	6 (9.8)	1 (1.8)		
Metastasis					0.674					0.015*	0.893
Single		67 (60.9)	19 (57.6)	48 (62.3)			70 (59.8)	43 (70.5)	27 (48.2)		
Multiple		43 (39.1)	14 (42.4)	29 (37.7)			47 (40.2)	18 (29.5)	29 (51.8)		
Metastasis resection		29 (26.4)	6 (18.2)	23 (29.9)	0.244		28 (23.9)	15 (24.6)	13 (23.2)	1.000	0.760
Total cycles of cetuximab^a^		8 (2–29)	9 (2–29)	8 (2–29)	0.537		–	–	–	–	–
Total cycles of chemotherapy^a^		13 (2–43)	13 (3–38)	13 (2–43)	0.928		13 (2–38)	14 (2–38)	13 (4–38)	0.546	0.344
All three active drugs^b^		73 (66.4)	24 (72.7)	49 (63.6)	0.388		89 (76.1)	48 (78.7)	41 (73.2)	0.522	0.109
Backbone chemotherapy					0.029*					0.041*	0.003*
Oxaliplatin-based		37 (33.6)	6 (18.2)	31 (40.3)			63 (53.8)	27 (44.3)	36 (64.3)		
Irinotecan-based		73 (66.4)	27 (81.8)	46 (59.7)			54 (46.2)	34 (55.7)	20 (35.7)		
Bevacizumab during the disease		44 (40.0)	15 (45.5)	29 (37.7)	0.525		38 (32.5)	24 (39.3)	14 (25.0)	0.116	0.270
Recurrent disease^c^		31 (28.2)	6 (18.2)	25 (32.5)	0.230		32 (27.4)	18 (29.5)	14 (25.0)	0.679	1.000

^a^The values are presented as median followed by range in parentheses; other values are presented as the number of patients followed by percentages in parentheses. ^b^ Patients who received 5-fluorouracil, oxaliplatin, and irinotecan during the course of their disease. ^c^ Patients who had metastatic disease after curative-intent primary tumor resection. ^d^ Patient characteristics were compared between the cetuximab and chemotherapy groups. ECOG, Eastern Cooperative Oncology Group; RSCC, right-sided colon cancer; LSCRC, left-sided colorectal cancer

**Table 2:** Baseline clinicopathologic characteristics of patients with second-line treatment

Clinicopathologic characteristic		Cetuximab group					Chemotherapy alone group				*P* value^c^
		All patients	RSCC	LSCRC	*P* value		All patients	RSCC	LSCRC	*P* value	
No. of patients		96	28	68	—		93	44	49	—	0.011*
Median age at diagnosis											
≤65 years		82 (85.4)	21 (75.0)	61 (89.7)	0.108		72 (77.4)	30 (68.2)	42 (85.7)	0.051	0.191
>65 years		14 (14.6)	7 (25.0)	7 (10.3)			21 (22.6)	14 (31.8)	7 (14.3)		
Sex					0.488					1.000	0.105
Male		63 (65.6)	20 (71.4)	43 (63.2)			50 (53.8)	24 (54.5)	26 (53.1)		
Female		33 (34.4)	8 (28.6)	25 (36.8)			43 (46.2)	20 (45.5)	23 (46.9)		
ECOG performance status					0.074					0.438	0.310
0		53 (55.2)	13 (46.4)	40 (58.8)			41 (44.0)	19 (43.2)	22 (44.9)		
1		35 (36.5)	11 (39.3)	24 (35.3)			42 (45.2)	22 (50.0)	20 (40.8)		
≥2		8 (8.3)	4 (14.3)	4 (5.9)			10 (10.8)	3 (6.8)	7 (14.3)		
Pathology					0.102					0.183	0.822
Adenocarcinoma		84 (87.5)	22 (78.6)	62 (91.2)			83 (89.2)	37 (84.1)	46 (93.9)		
Mucinous & signet-ring cell		12 (12.5)	6 (21.4)	6 (8.8)			10 (10.8)	7 (15.9)	3 (6.1)		
Metastasis					0.804					1.000	0.005*
Single		69 (71.9)	21 (75.0)	48 (70.6)			48 (51.6)	23 (52.3)	25 (51.0)		
Multiple		27 (28.1)	7 (25.0)	20 (29.4)			45 (48.4)	21 (47.7)	24 (49.0)		
Metastasis resection		24 (25.0)	6 (21.4)	18 (26.5)	0.796		16 (17.2)	11 (25.0)	15 (30.6)	0.646	0.215
Total cycles of cetuximab (median)		5 (2-20)	4 (2-13)	5 (2-20)	0.272		—	—	—	—	—
Total cycles of chemotherapy (median)		8 (2-66)	7 (4-35)	8 (2-66)	0.548		6 (2-36)	6 (2-36)	6 (2-27)	1.000	0.031*
All three active drugs^a^		87 (90.6)	24 (85.7)	63 (92.6)	0.441		84 (90.3)	39 (88.6)	45 (91.8)	0.731	1.000
Backbone chemotherapy					0.139					1.000	0.745
Oxaliplatin-based		27 (28.1)	11 (39.3)	16 (23.5)			24 (25.8)	11 (25.0)	13 (26.5)		
Irinotecan-based		69 (71.9)	17 (60.7)	52 (76.5)			69 (74.2)	33 (75.0)	36 (73.5)		
Bevacizumab during the disease		27 (28.1)	11 (39.3)	16 (23.5)	0.637		22 (23.7)	8 (18.2)	14 (28.6)	0.329	0.510
Recurrent disease^b^		42 (43.8)	6 (21.4)	36 (52.9)	0.006*		33 (35.5)	19 (43.2)	14 (28.6)	0.199	0.298

All values are presented as the number of patients followed by percentages in parentheses. ^a^ Patients who received 5-fluorouracil, oxaliplatin, and irinotecan during the course of their disease. ^b^ Patients who had metastatic disease after curative-intent primary tumor resection. ^c^ Patient characteristics were compared between the cetuximab and chemotherapy groups. Other footnotes as in Table 1

For the first-line therapy, more patients in the chemotherapy alone group received oxaliplatin-based chemotherapy than those in the cetuximab group (53.8 % vs. 33.6 %, *P* = 0.003). By contrast, in those who received second-line therapy, more patients in the cetuximab group had single organ metastasis (71.9 % vs. 51.6 %, *P* = 0.005) than those in the chemotherapy alone group. In addition, patients in the cetuximab group had a longer duration of chemotherapy administration as second-line therapy (8 vs. 6 cycles, *P* = 0.031) than those in the chemotherapy alone group.

Patients were separated into two subgroups (RSCC and LSCRC) according to the primary tumor location. In the first-line therapy subgroup (Table 1), age, sex, Eastern Cooperative Oncology Group (ECOG) performance status, tumor grade, location of metastasis, resection of the primary or metastatic site, total cycles of cetuximab or chemotherapy, percentage of three active chemotherapy drugs (5-fluorouracil [FU], oxaliplatin, and irinotecan), treatment with bevacizumab, and recurrent disease did not differ between patients with RSCC and those with LSCRC in both the cetuximab group and the chemotherapy alone group. The proportion of mucinous or signet-ring cell carcinomas was significantly higher in patients with RSCC than in patients with LSCRC in the cetuximab group (21.2 % vs. 2.6 %, *P* = 0.003). The proportion of patients with multiple metastases was significantly higher in patients with LSCRC than in those with RSCC in the chemotherapy alone group (51.8 % vs. 29.5 %, *P* = 0.015). More patients with RSCC received irinotecan-based first-line chemotherapy than patients with LSCRC in either the cetuximab group (81.8 % vs. 59.7 %, *P* = 0.029) or in the chemotherapy alone group (55.7 % vs. 35.7 %, *P =* 0.041)

### ORR

As a first-line treatment, the addition of cetuximab to chemotherapy significantly improved the ORR compared with chemotherapy alone in patients with LSCRC (49.4 % vs. 28.6 %, *P* = 0.005) (Table 3). However, the ORR was comparable between the two groups in patients with RSCC (36.4 % vs. 26.2 %, *P* = 0.349). There was no significant difference in the response rate between patients with LSCRC and those with RSCC in both the chemotherapy alone group (28.6 % vs. 26.2 %, *P* = 0.837) and the cetuximab group (49.4 % vs. 36.4 %, *P* = 0.296) (Tables 4 and 5, respectively).

**Table 3:** Objective response rate to first-line treatment in patients with metastatic colorectal cancer

Response	RSCC		LSCRC	
	Chemotherapy alone group	Cetuximab group	Chemotherapy alone group	Cetuximab group
Total	61	33	56	77
Best ORR	16 (26.2)	12 (36.4)	16 (28.6)	38 (49.4)
CR	3 (4.9)	0 (0)	0 (0)	1 (1.3)
PR	13 (21.3)	12 (36.4)	16 (28.6)	37 (48.1)
SD	28 (45.9)	11 (33.3)	28 (50.0)	33 (42.8)
PD	14 (23.0)	10 (30.3)	12 (21.4)	4 (5.2)
NA	3 (4.9)	0 (0)	0 (0)	2 (2.6)
*P* value ^a^	0.349		0.005	

All values are presented as the number of patients followed by the percentages in parentheses

^a^ The ORR to the first-line treatment was compared between the chemotherapy and cetuximab plus chemotherapy groups in RSCC and LSCRC patients, respectively

ORR, objective response rate; CR, complete response; PR, partial response; SD, stable disease; PD, progressive disease; NA, not available. Other footnotes as in Table 1

**Table 4:** Objective response rates to first- and second-line treatments in patients who received chemotherapy alone

Response	Fist-line treatment		Second-line treatment	
	RSCC	LSCRC	RSCC	LSCRC
Total	61	56	44	49
Best ORR	16 (26.2)	16 (28.6)	5 (11.4)	5 (10.2)
CR	3 (4.9)	0 (0)	0 (0)	0 (0)
PR	13 (21.3)	16 (28.6)	5 (11.4)	5 (10.2)
SD	28 (45.9)	28 (50.0)	21 (47.7)	23 (46.9)
PD	14 (23.0)	12 (21.4)	16 (36.4)	19 (38.8)
NA	3 (4.9)	0 (0)	2 (4.5)	2 (4.1)
*P* value^a^	0.837		1.000	

^a^ The ORR was compared between the RSCC and LSCRC patients who received chemotherapy alone. Footnotes as in Tables 1 and 3

**Table 5:** Objective response rates of first- and second-line treatments in patients who received cetuximab plus chemotherapy

Response	Fist-line treatment		Second-line treatment	
	RSCC	LSCRC	RSCC	LSCRC
Total	33	77	28	68
Best ORR	12 (36.4)	38 (49.4)	2 (7.1)	16 (23.5)
CR	0 (0)	1 (1.3)	0 (0)	1 (1.5)
PR	12 (36.4)	37 (48.1)	2 (7.1)	15 (22.0)
SD	11 (33.3)	33 (42.8)	16 (57.2)	34 (50.0)
PD	10 (30.3)	4 (5.2)	10 (35.7)	17 (25.0)
NA	0 (0)	2 (2.6)	0 (0)	1 (1.5)
*P* value^a^	0.296		0.085	

^a^ The ORR was compared between RSCC and LSCRC patients who received chemotherapy alone. Other footnotes as in Tables 1 and 3

As a second-line treatment, cetuximab with chemotherapy had a tendency to improve the ORR compared with chemotherapy alone in patients with LSCRC (23.5 % vs. 7.1 %, *P* = 0.085), but not in those with RSCC (7.1 % vs. 11.4 %, *P* = 0.698) (Table 6). Although there was no difference in response rate between patients with LSCRC and RSCC treated with chemotherapy alone (10.2 % vs. 11.4 %, *P* = 1.000), cetuximab with chemotherapy exhibited a trend of improving response rate in patients with LSCRC compared with those with RSCC (23.5 % vs. 7.1 %, *P* = 0.085) (Tables 4 and 5, respectively).

**Table 6:** Objective response rates to second-line treatment in patients with metastatic colorectal cancer

Response	RSCC		LSCRC	
	Chemotherapy alone group	Cetuximab group	Chemotherapy alone group	Cetuximab group
Total	44	28	49	68
Best ORR	5 (11.4)	2 (7.1)	5 (10.2)	16 (23.5)
CR	0 (0)	0 (0)	0 (0)	1 (1.4)
PR	5 (11.4)	2 (7.1)	5 (10.2)	15 (22.1)
SD	21 (47.7)	16 (57.2)	23 (46.9)	34 (50.0)
PD	16 (36.4)	10 (35.7)	19 (38.8)	17 (25.0)
NA	2 (4.5)	0 (0)	2 (4.1)	1 (1.5)
*P* value ^a^	0.698		0.087	

^a^ The ORR to the second-line treatment was compared between the chemotherapy and cetuximab plus chemotherapy groups of RSCC and LSCRC patients, respectively.

Other footnotes as in Tables 1 and 3

### PFS and OS

**Fig. 1:**
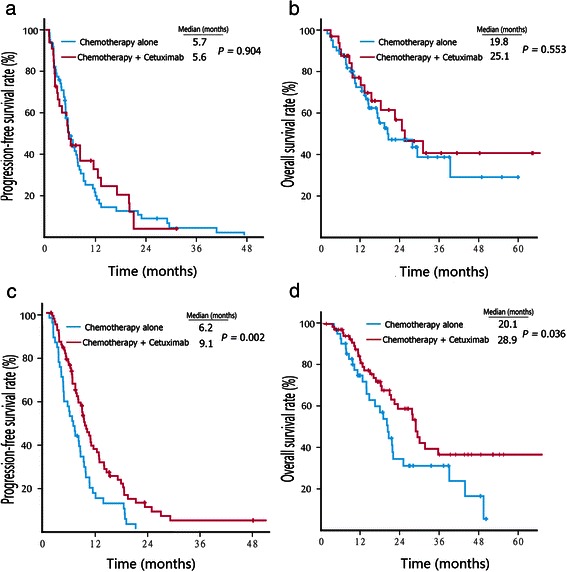
Kaplan–Meier survival estimates of metastatic colorectal cancer patients with first-line therapy. **a**, progression-free survival (PFS) curves of right-sided colon cancer (RSCC) patients. **b**, overall survival (OS) curves of RSCC patients. **c**, PFS curves of left-sided colorectal cancer (LSCRC) patients. **d**, OS curves of LSCRC patients

In RSCC patients who received first-line chemotherapy, the median PFS (5.6 vs. 5.7 months, *P* = 0.904) (Fig. 1a) and OS (25.1 vs. 19.8 months, *P* = 0.553) (Fig. 1b) were comparable between those who received cetuximab with chemotherapy and chemotherapy alone. On the other hand, first-line treatment with cetuximab plus chemotherapy resulted in a significantly longer median PFS (9.1 vs. 6.2 months, *P* = 0.002) (Fig. 1c) and OS (28.9 vs. 20.1 months, *P* = 0.036) (Fig. 1d) compared with chemotherapy alone in patients with LSCRC.

**Fig. 2:**
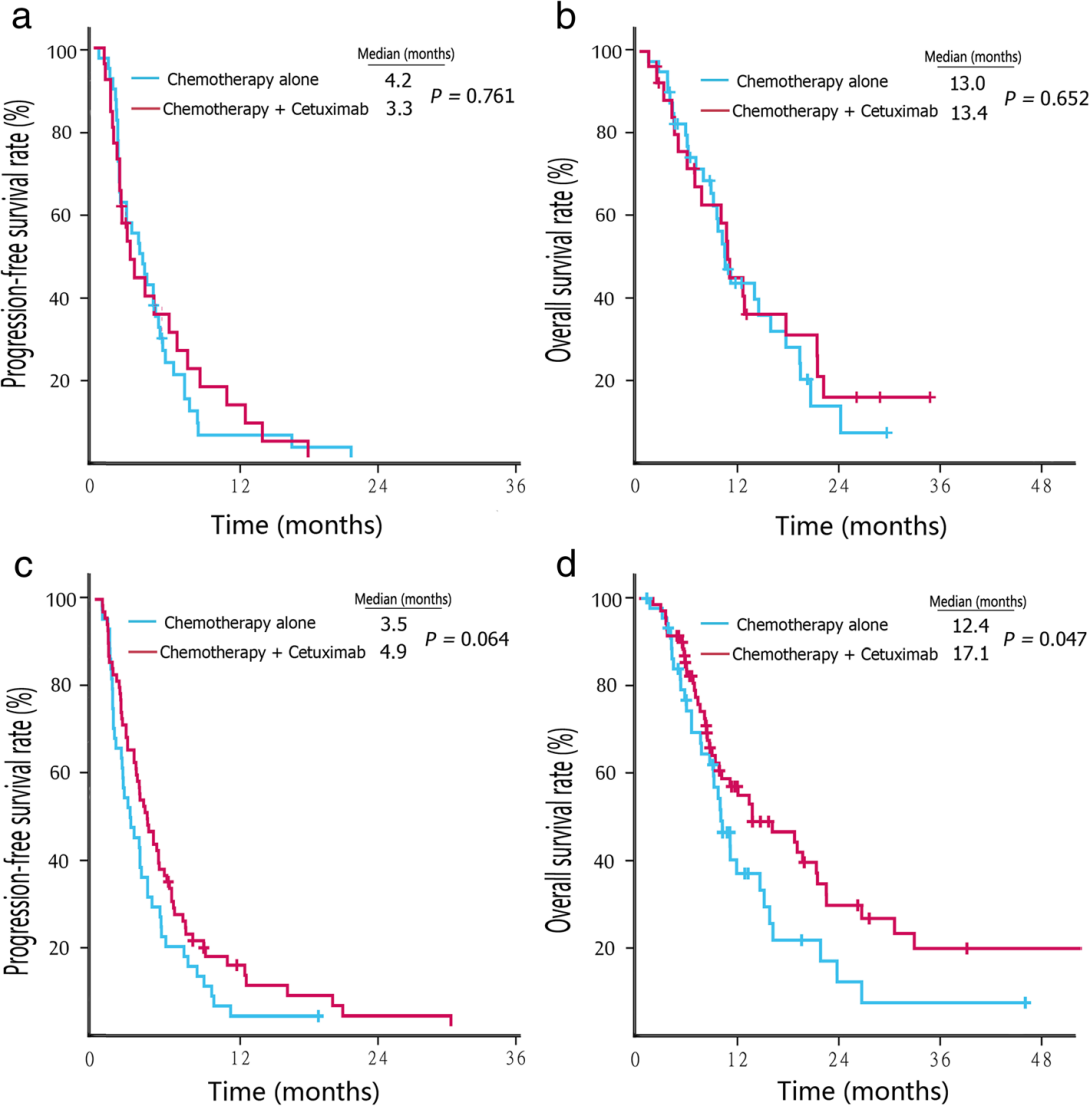
Kaplan–Meier survival estimates of metastatic colorectal cancer patients with second-line therapy. **a**, PFS curves of RSCC patients. **b**, OS curves of RSCC patients. **c**, PFS curves of LSCRC patients. **d**, OS curves of LSCRC patients

For second-line therapy, the addition of cetuximab treatment did not prolong the median PFS (3.3 vs. 4.2 months, *P* = 0.761) (Fig. 2a) or OS (13.4 vs. 13.0 months, *P* = 0.652) (Fig. 2b) in patients with RSCC. However, in patients with LSCRC, the combination of cetuximab with chemotherapy had a tendency to prolong the median PFS (4.9 vs. 3.5 months, *P* = 0.064) (Fig. 2c), and it significantly prolonged the OS (17.1 vs. 12.4 months, *P* = 0.047) (Fig. 2d).

**Fig. 3:**
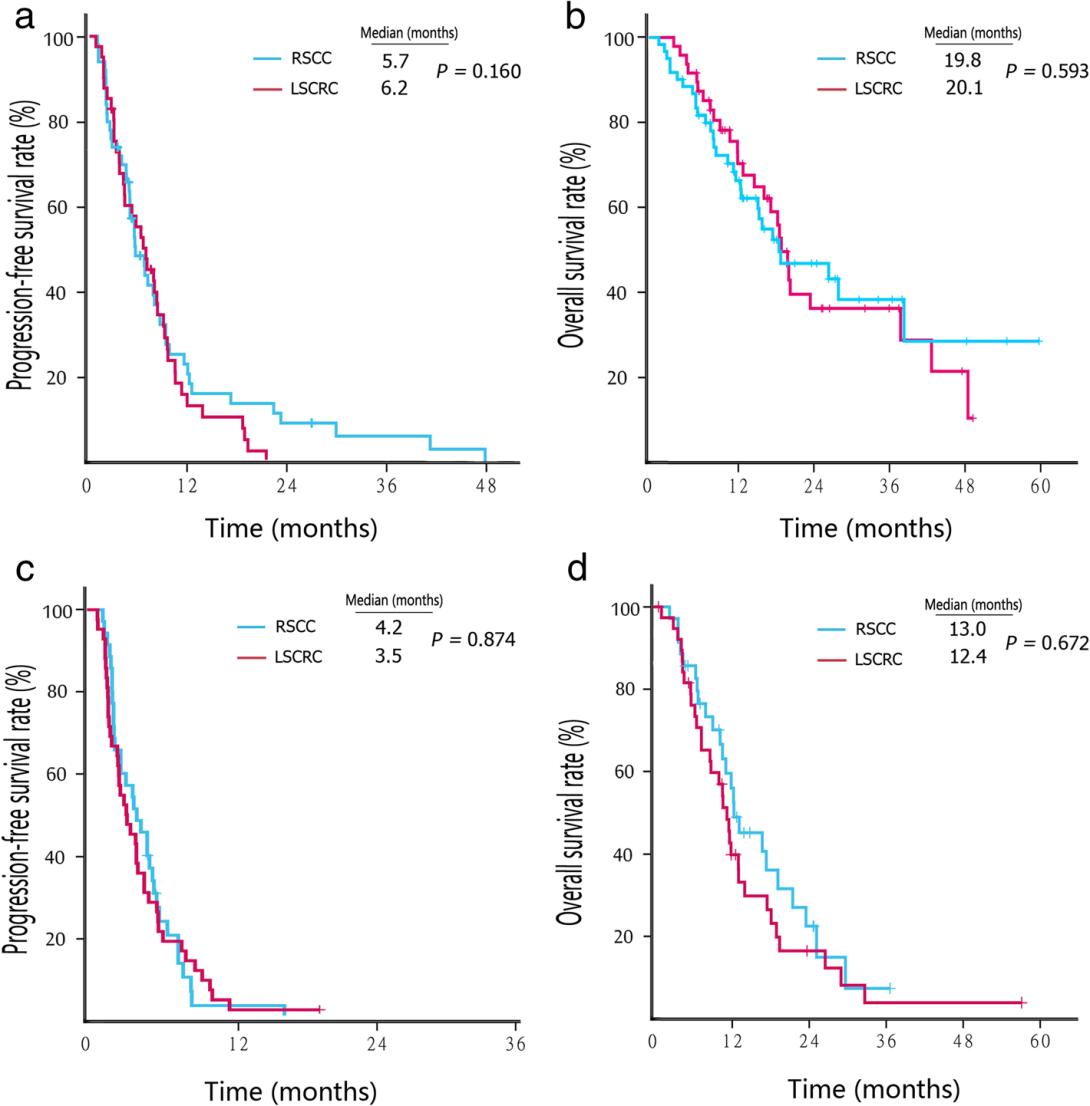
Kaplan–Meier survival estimates of metastatic colorectal cancer patients treated with chemotherapy alone. **a**, PFS curves of patients who received first-line therapy. **b**, OS curves of patients who received first-line therapy. **c**, PFS curves of patients who received second-line therapy. **d**, OS curves of patients who received second-line therapy

**Fig. 4:**
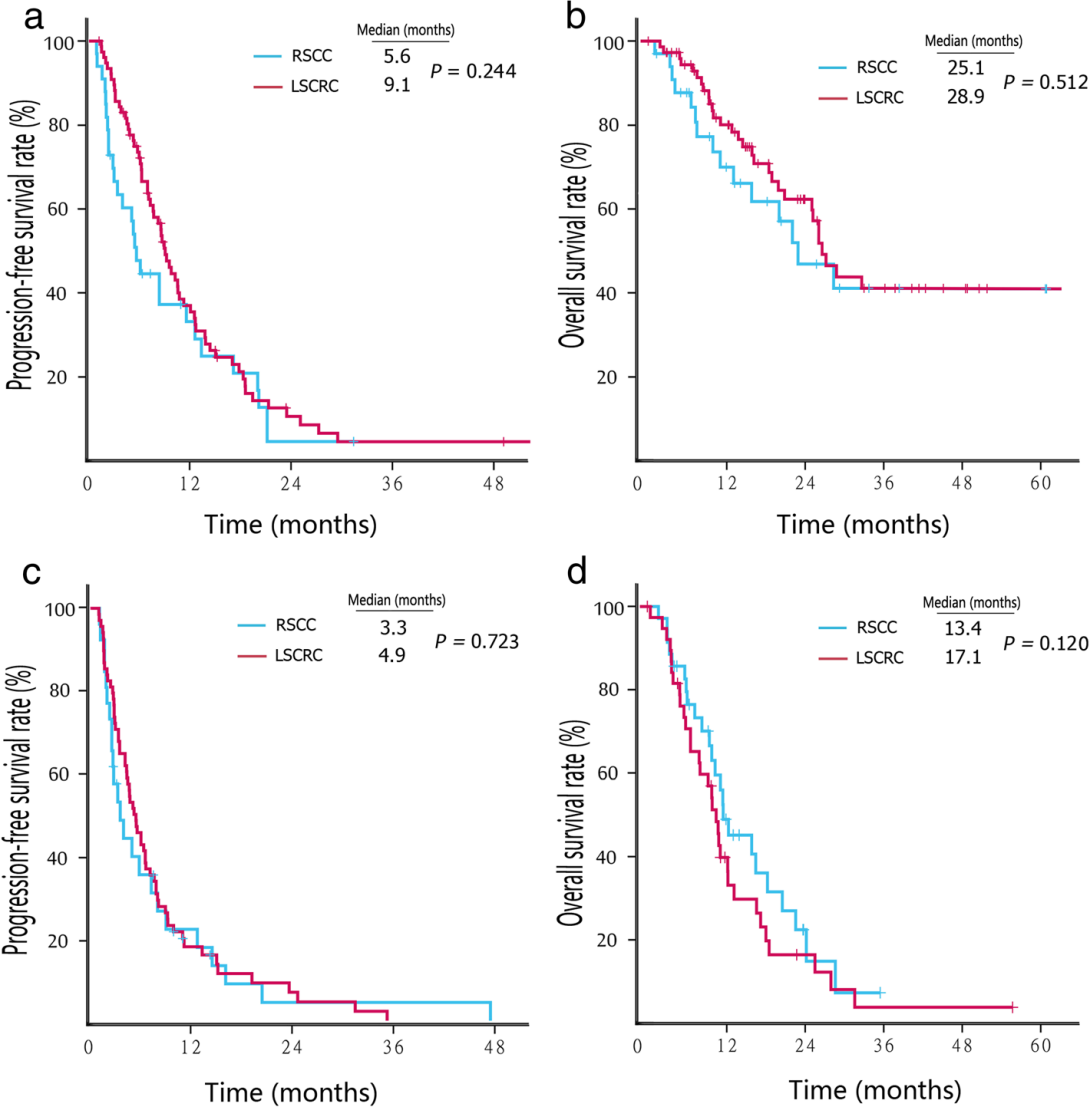
Kaplan–Meier survival estimates of metastatic colorectal cancer patients treated with cetuximab plus chemotherapy. **a**, PFS curves of patients who received first-line therapy. **b**, OS curves of patients who received first-line therapy. **c**, PFS curves of patients who received second-line therapy. **d**, OS curves of patients who received second-line therapy

We also compared the PFS and OS of patients undergoing chemotherapy alone between RSCC and LSCRC groups. No difference in survival was observed between the RSCC and LSCRC patients who received chemotherapy alone as a first-line (PFS, 5.7 vs. 6.2 months, *P* = 0.160; OS, 19.8 vs. 20.1 months, *P* = 0.593; Fig. 3a and b) or second-line therapy (PFS, 4.2 vs. 3.5 months, *P* = 0.874; OS, 13.0 vs. 12.4 months, *P* = 0.672; Fig. 3c and d). Similarly, no difference in survival was observed between the RSCC and LSCRC patients who received cetuximab combined with chemotherapy as a first-line (PFS, 5.6 vs. 9.1 months, *P* = 0.244; OS, 25.1 vs. 28.9 months, *P* = 0.512; Fig. 4a and b) or second-line therapy (PFS, 3.3 vs. 4.9 months, *P* = 0.723; OS, 13.4 vs. 17.1 months, *P* = 0.120; Fig. 4c and d).

## Discussion

The current study showed a significant difference in the ORR and OS between patients with metastatic LSCRC and RSCC who underwent first- or second-line cetuximab treatment. Patients with metastatic LSCRC exhibited a higher response rate to cetuximab plus chemotherapy than to chemotherapy alone, and cetuximab significantly prolonged the PFS and OS in these patients. However, this improvement in the OS or PFS was not observed in patients with RSCC who were treated with cetuximab.

The difference in the response to chemotherapy between LSCRC and RSCC groups was previously reported. However, the current study did not observe a different response to chemotherapy between patients with LSCRC and RSCC. The combination of cetuximab with chemotherapy had a tendency to improve the response rate in LSCRC patients compared with RSCC patients. One study reported that men with right-sided Duke’s C colon cancer benefited from adjuvant chemotherapy, but men with left-sided tumors did not [24]. The study also found a significantly higher frequency of MSI in right-sided than in left-sided tumors (20 % vs. 1 %). In the AIO KRK-0104 trial which investigated first-line therapy for mCRC with cetuximab, capecitabine, and irinotecan versus cetuximab, capecitabine, and oxaliplatin, LSCRC was associated with a significantly longer OS and PFS compared with RSCC [25]. A recent study of pre-treated, chemotherapy-refractory mCRC patients showed that cetuximab was associated with a longer PFS compared with best support care in patients with LSCRC (5.4 vs. 1.8 months, *P* < 0.001), but not in those with RSCC (1.9 vs. 1.9 months, *P* = 0.26) [26].

Although the current study observed an interesting phenomenon, it needs to be confirmed in a randomized prospective study. The underlying mechanisms for the differences in the response rates between LSCRC and RSCC remain unclear. Because the benefit of cetuximab is limited to mCRC patients with wild-type *KRAS* tumors, the *KRAS* status in both LSCRC and RSCC needs to be taken into consideration [27]. Several studies found a higher rate of *KRAS* mutations in RSCC than that in LSCRC [14, 15]. Other studies found similar *KRAS* mutation rates in both RSCC and LSCRC [8, 16]. In addition to *KRAS*, the *BRAF* V600E mutation negatively impacts the treatment outcomes for cetuximab in mCRC patients [23]. The *BRAF* mutation is observed more frequently in RSCC than in LSCRC, which might partly explain the higher response rate to cetuximab in patients with LSCRC [16, 28]. However, the *NRAS* mutation is mainly found in LSCRC [29]. Alterations of other molecules, including EGFR polysomy, EGFR amplification, and phosphatase and tensin homolog (PTEN) null expression, have been found to be associated with the outcome of cetuximab treatment [30].

## Conclusions

In conclusion, the current study suggests that the primary tumor location may affect the response of patients with mCRC to cetuximab. The addition of cetuximab to first-line and second-line chemotherapy may only benefit patients with metastatic LSCRC. These results should be further confirmed in a prospective study.
